# Emphysematous cystitis: An unusual disease of the Genito-Urinary system suspected on imaging

**DOI:** 10.1186/1476-0711-3-20

**Published:** 2004-10-05

**Authors:** Ravi K Bobba, Edward L Arsura, Pawanjit S Sarna, Amar K Sawh

**Affiliations:** 1 Department of Internal Medicine Veterans Affairs Medical Center University of Virginia, Roanoke-Salem Program, Virginia, USA

**Keywords:** hematuria, emphysematous cystitis

## Abstract

Emphysematous cystitis is a rare disease entity caused by gas fermenting bacterial and fungal pathogens. Clinical symptoms are nonspecific and diagnostic clues often arise from the unanticipated imaging findings. We report a case of 52-year-old male who presented with fever, dysuria and gross hematuria who was found to have emphysematous cystitis.

## Introduction

Emphysematous cystitis is an uncommon, but severe manifestation of infection of the urinary bladder produced by gas forming organisms. The presentation may be atypical and contrary to the degree of inflammation, patients may present with subtle clinical findings. A high index of suspicion, especially in susceptible populations, is needed. We report a case of middle-aged, poorly controlled, diabetic male who presented with dysuria, fever and hematuria and was found to have *Escherichia coli *emphysematous cystitis that resolved with antibiotic treatment.

## Case Report

A 52-year-old male presented with fever, chills and blood in the urine. Four days prior to admission, he noted increased urinary frequency, urgency, occasional incontinence and burning sensation during micturition. His past medical history is significant for coronary artery disease, hyperlipidemia, hypertension and insulin dependent diabetes mellitus (most recent HgbA1c of 11.7 two weeks prior). He had 66-pack year history of smoking. Medications on admission included, aspirin, atenolol, cyclobenzaprine, gabapentin, gemfibrozil, glipizide, NPH insulin, hydrochlorthiazide/triamterene, nifedipine, nitroglycerin patch, omeprazole, and sertraline.

On physical examination, his temperature was 100.9°F, pulse 88, BP 123/71 mm/hg. Cardiac and pulmonary examinations were unremarkable. His abdomen was soft, nontender with positive bowel sounds and no organomegaly detected. The rectal examination was normal with a non-tender, smooth prostate. Neurological exam was exam was unremarkable.

**Figure 1:**
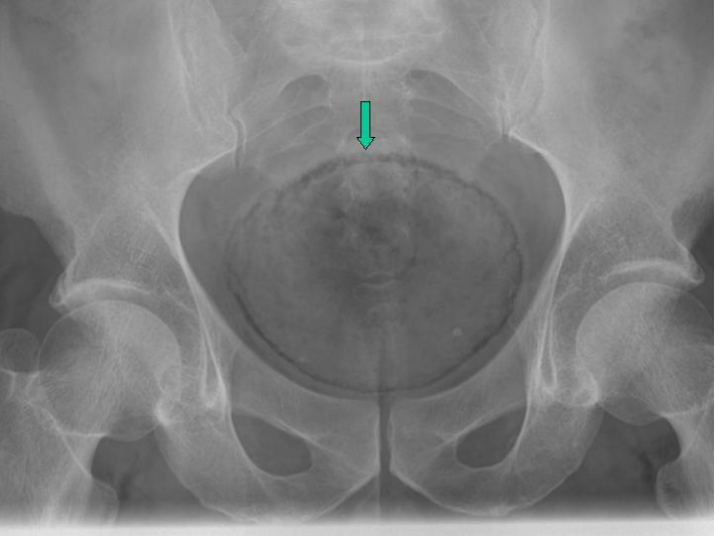
X-ray of pelvis showing gas in the urinary bladder wall (Arrow)

**Figure 2:**
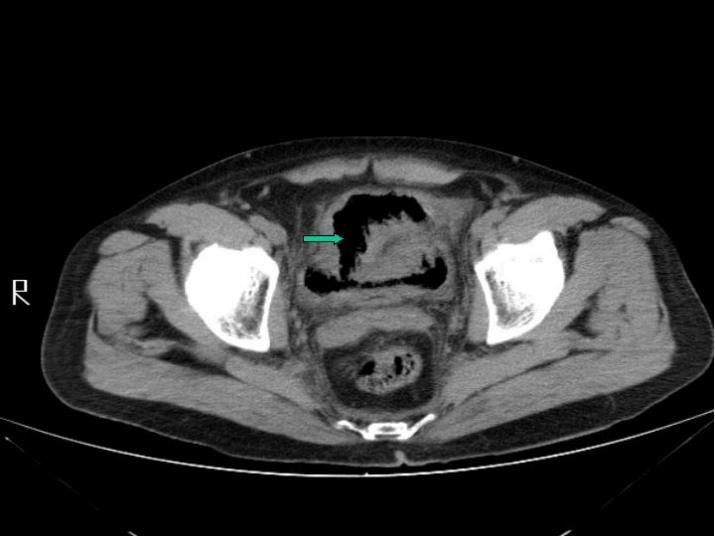
CT scan of the pelvis revealing gas in the bladder and the bladder wall.

Laboratory evaluation revealed white cell count 17,400 cells/mm^3^, hemoglobin 15.1 gm/dl, sodium 128 mmol/l, potassium 4.1 mmol/l, chloride 91 mmol/l, bicarbonate 24 mmol/L, glucose 273 mg/dl and an anion gap of 13. Urine analysis revealed red, cloudy urine with pH 5.0, nitrite positive and numerous white and red blood cells on microscopy. A pelvic X-ray (figure 1) showed circumferential air in the bladder wall. CT scan of the pelvis (figure 2) revealed gas in the bladder lumen and wall extending to the right ureter. He was empirically treated with gentamicin and piperacillin/tazobactam. He responded with defervescence and a decline in his white cell count. Urine cultures grew *Escherichia coli *and based on the sensitivities he was discharged home on ciprofloxacin.

## Discussion

Emphysematous cystitis is a rare entity characterized by pockets of gas in and around the bladder wall produced by bacterial or fungal fermentation [1,2]. Patients may complain of irritative symptoms, abdominal discomfort or pneumaturia. A history of pneumaturia is highly suggestive, but is rarely offered by the patient. As occurred in our case and in a number of cases in the literature, the clinical features were inconclusive or actually unhelpful [3-6]. The radiographic findings provided the first and only diagnostic clue. The disease is often associated with female sex, immunocompromised state, diabetes mellitus, previous recurrent urinary tract infections, urinary stasis, neurogenic bladder and in transplant recipients [7]. Therefore, in susceptible patients, with the above risk factors along with signs and symptoms of urinary tract infection, the index of suspicion for this entity should be high. The most common organism is *E. coli *[2], but other organisms reported to produce emphysematous cystitis include *Enterobacter aerogenes*, *Klebsiella pneumonia*, *Proteus mirabilis*, *Staphylococcus aureus*, *streptococci*, *Clostridium perfringens *[8], *and Candida albicans *[9]. The mechanism by which gas appears in the wall of the bladder may involve either transluminal dissection of gas or true infection of the bladder wall with pathogens.

Diagnostic entities associated with gas in the genitourinary tract include emphysematous pyelonephritis, emphysematous pyelitis, and gas-forming renal abcess. Patients with emphysematous cystitis are not as acutely ill as those with pyelonephritis or pyelitis. Abdomino-pelvic CT scan can further delineate the extent of disease. It is important to differentiate emphysematous cystitis from emphysematous pyelonephritis, in which gas involves the renal parenchyma, since the latter has an increased mortality and generally requires nephrectomy. In contrast surgical intervention is rarely needed in emphysematous cystitis except when an anatomical abnormality like an obstruction or stone is present [10].

The source of this gas within the urinary tract is from infection, trauma, vesico-enteric fistulas from radiation therapy, rectal carcinoma, diverticular disease or Crohn's disease and iatrogenic causes, such as diagnostic or surgical instrumentation. History, physical exam and imaging are the best modalities to differentiate the above etiologic causes. Fistulous tracts, abscess, can be excluded on CT scan.

Emphysematous cystitis requires aggressive treatment with parenteral antibiotics and bladder drainage [11]. Delayed diagnosis may lead to unfavorable outcomes including overwhelming infection, extension to ureters and renal parenchyma, bladder rupture and death. Improved outcomes may be achieved by early recognition of the infection, by clinical and radiological assessment, and by appropriate antibiotic therapy.

## Conclusion

Emphysematous cystitis most often is not diagnosed by routine or systematic approach. It is a rare entity, detected on imaging, and the physician should be cautious, tailor the diagnostic approach to individual patients based on the suspicion, available clinical and radiological data, and consider emphysematous cystitis in the differential diagnosis of hematuria in a patient with known risk factors.
